# Books and Magazines

**Published:** 1903-05

**Authors:** 


Disinfection and Disinfectants.—A Practical Guide for Sanitarians, Health and Quarantine Officers. By M. J. Rosenau, M. D., Director of the Hygienic Laboratory and Passed Assistant-Surgeon U. S. Public Health and Marine Hospital Service, Washington, D. C. Illustrated. Cloth. Pp., 353. Price, $2.00, net. Philadelphia: P. Blakiston's Sons & Co. 1902.

Dr. Rosenau has furnished a convenient manual of disinfection which can not fail to be of use to physicians, health officers and others, in fact, to everyone who has occasionally the need of such a work. He says he has made mistakes like everyone else in learning what, how and when to disinfect, and the book is written to aid others who have to battle with infection and communicable diseases. It is based on experience as well as knowledge gained otherwise and has, therefore, its special value as a work of a practical expert. It is a book the substance of which ought to be familiar to every physician, and especially to every local health officer in the country.

---

Elementary Studies in Insect Life.—By S. J. Hunter, Associate Professor of Comparative Zoology and Entomology, in the University of Kansas, presents the biologic phases of insect life on a new plan. It begins with the earliest stages of insect growth and development, and leads the student up to some of the more important phases of biology as presented by insects. The book assists and encourages the student to learn from independent personal observation, such facts as he can in field and laboratotry concerning insect life.

While this work comes under the head of text-books, there is much in it of value to the general reader. The relations which insects bear to man's welfare are clearly and intelligently set forth. The farmer and fruit-grower will appreciate the story of their friends and foes among the insect tribe. The illustrations are a feature of the book worthy of special comment. Those interested in the camera and outdoor photography will place high value on this book by reason of the methods used and results obtained in procuring many of these photographs from life. The work is 12 mo. in size, has 369 pages, two colored plates, and 260 illustrations. Crane & Co., Topeka, Kans. Price, $1.25, postpaid.

